# An experimental study of Quartets MaxCut and other supertree methods

**DOI:** 10.1186/1748-7188-6-7

**Published:** 2011-04-19

**Authors:** M Shel Swenson, Rahul Suri, C Randal Linder, Tandy Warnow

**Affiliations:** 1 Department of Computer Science, The University of Texas at Austin, Austin TX, USA; 2 Section of Integrative Biology, The University of Texas at Austin, Austin TX, USA

## Abstract

**Background:**

Supertree methods represent one of the major ways by which the Tree of Life can be estimated, but despite many recent algorithmic innovations, matrix representation with parsimony (MRP) remains the main algorithmic supertree method.

**Results:**

We evaluated the performance of several supertree methods based upon the Quartets MaxCut (QMC) method of Snir and Rao and showed that two of these methods usually outperform MRP and five other supertree methods that we studied, under many realistic model conditions. However, the QMC-based methods have scalability issues that may limit their utility on large datasets. We also observed that taxon sampling impacted supertree accuracy, with poor results obtained when all of the source trees were only sparsely sampled. Finally, we showed that the popular optimality criterion of minimizing the total topological distance of the supertree to the source trees is only weakly correlated with supertree topological accuracy. Therefore evaluating supertree methods on biological datasets is problematic.

**Conclusions:**

Our results show that supertree methods that improve upon MRP are possible, and that an effort should be made to produce scalable and robust implementations of the most accurate supertree methods. Also, because topological accuracy depends upon taxon sampling strategies, attempts to construct very large phylogenetic trees using supertree methods should consider the selection of source tree datasets, as well as supertree methods. Finally, since supertree topological error is only weakly correlated with the supertree's topological distance to its source trees, development and testing of supertree methods presents methodological challenges.

## Background

Because of the computational difficulties in estimating large phylogenies, many computational biologists think that the only feasible strategy to estimating the Tree of Life will involve a divide-and-conquer approach where trees are estimated on subsets of taxa and a computational method is used to assemble a tree on the entire taxon set from these smaller trees. These methods are called *supertree methods*, the smaller trees are called *source trees *and the set of these source trees is called a *profile *of trees. While there are many supertree methods, only matrix representation with parsimony (MRP) [1,2] is used regularly in supertree constructions on biological datasets [3,4].

Quartet amalgamation methods (methods that construct supertrees when all source trees are four-leaf trees) can also be used as generic supertree methods, as follows. First, each estimated source tree is replaced with a subset of its induced quartet trees, and then the sets of quartet trees are combined into a collection of quartet trees (some from each source tree). This set is then passed to the quartet amalgamation method to estimate a supertree.

Constructing a tree from a set of quartet trees presents computational challenges. For example, the natural optimization problem, Maximum Quartet Consistency (MQC), in which the input is a set of quartet trees and a supertree is sought that displays the maximum number of quartet trees, is NP-hard, and generally hard to approximate except in special cases [5-8]. Theoretical results and heuristics for the special case where the input set contains a tree on every quartet appear in [9-13].

In a recent paper [14], Snir and Rao presented *Quartets MaxCut *(QMC), a heuristic for MQC that can be applied to arbitrary sets of quartet trees (i.e., ones that may not contain a tree on every quartet). Snir and Rao showed that by encoding the source trees as quartet trees, QMC could be used as a supertree method for arbitrary inputs. Their study evaluated this QMC-based supertree method for a number of biological supertree profiles; however, since the true supertree was not known, they could not evaluate the topological accuracy of the supertrees they constructed. Instead, they computed the average similarity of the QMC and MRP supertrees to the source trees, using two different similarity measures. This comparison showed that QMC had higher average similarity to the source trees under one criterion, and lower average similarity with respect to another; thus, Snir and Rao failed to establish that QMC produced "better" trees than MRP.

QMC's failure to outperform MRP as a supertree method with respect to the supertrees' average similarity to the source trees should not be considered a serious problem for the QMC method for two reasons. First, average similarity to the source trees is not the same as accuracy with respect to the true tree (a question we investigate directly in this paper). Second, QMC depends critically upon the specific technique used to encode each source tree as a set of quartet trees. Therefore, QMC might be producing highly accurate supertrees even though their average similarity to their source trees is lower than MRP supertrees, and it might be capable of producing more accurate supertrees if other encodings of the source trees were used. In this paper, we report results from a study in which we explored several encodings of the source trees by quartet trees and applied QMC to the resultant sets of quartet trees. We compared these different QMC-based supertree methods to MRP and five other supertree methods: Robinson-Foulds Supertrees (RFS) [15], Q-imputation (Q-Imp) [16], MinFlip [17-19], SFIT [20], and PhySIC [21]. We find:

• The topological accuracy of QMC supertrees computed from different encodings varied substantially.

• Two QMC-based supertree methods, QMC(All) and QMC(Exp+TSQ) (differing only in how the source trees are encoded) produced more accurate supertrees than all the other supertree methods under many realistic model conditions, and had comparable accuracy under most others. However, both of these QMC-based supertree methods had problems with profiles containing large source trees. For such profiles, QMC(All) often failed to run, and QMC(Exp+TSQ) performed less well than MRP. Finally, when both QMC methods could be run their results were comparable.

• Supertrees estimated on profiles in which all the source trees were based upon sparsely sampled taxa tended to have poor accuracy by comparison to supertrees estimated on profiles in which most source trees were clade-focused. Therefore, the taxon sampling strategies used to define the source tree datasets impacts supertree accuracy, and needs to be considered in the design of supertree studies.

• Topological similarity of supertrees to their source trees is not strongly correlated with topological accuracy of supertrees. Thus, evaluating supertree methods on biological datasets is problematic, and supertree methods that seek to minimize topological distance to their source trees may not have the best accuracy.

## Methods

### Basics

#### Supertree datasets

Because of the taxon sampling strategies used by biologists, source trees tend to be focused either on intensively sampled, smaller subgroups, like big cats, or on larger, sparsely sampled groups, like all vertebrates. We refer to the first type as a *clade-based *source tree, and the second type as a *scaffold*. Supertree profiles include scaffolds to ensure sufficient overlap with the clade-based trees.

#### Matrix representation with parsimony

MRP encodes source trees as a matrix of *partial binary characters: *all entries in the matrix are 0, 1, or ?, with each column in the matrix defined by a single edge in a source tree. The matrix is then analyzed using a heuristic for the NP-hard maximum parsimony problem [22].

#### Quartets MaxCut (QMC)

QMC is a quartet amalgamation method, operating in polynomial time and providing no guarantees with respect to its optimization problem, MQC. The source trees are encoded by sets of quartet trees, and QMC is applied to the union of these sets.

#### Quartet encodings of source trees

The work presented here explored several techniques for representing source trees by sets of quartet trees. Two of these techniques use random sampling strategies [14], which are based upon computation of the topological distance between leaves in the source tree. The *topological diameter *of a quartet tree *q *with respect to a source tree *t *(denoted *diam_t_*(*q*)) is the maximum of its leaf-to-leaf topological distances within *t*. The quartet encoding strategies used in [14] also included calculation of the *Topologically-Short Quartet *(TSQ) trees, defined as follows. For each edge in a source tree, pick the topologically nearest leaves in each of the subtrees around the edge. If two or more leaves within a subtree have the same topological distance to the edge, pick all such leaves. The set of quartet trees formed by picking one such leaf from each subtree forms the TSQs around that edge. The union of all these is the set of TSQ trees.

We tested three strategies for encoding a source tree *t *by a set of quartet trees:

**All quartets**: include all induced four-taxon trees.

**Geo+TSQ**: include a quartet *q *with probability *d***^-^**^3^where *d *= *diam_t_*(*q*), and add the TSQ trees (this method was studied in [14]).

**Exp+TSQ**: compute the topological distance between every pair of leaves, include a quartet with probability 1.5^-*d *^where *d *= *diam_t_*(*q*), and add the TSQ trees (this method was also studied in [14]).

### Performance study design

Our simulation study used datasets that have properties typical of biological supertree datasets, and that were used in a previous study [23] to compare supertree methods to combined analysis using maximum likelihood. These datasets had 100, 500 and 1000 taxa, and came in two types: (1) *mixed *source trees, consisting of one scaffold dataset (produced by a random selection of taxa from the entire dataset) and many clade-based datasets (focused dense taxon sampling within a rooted subtree), and (2) *all-scaffold *source trees, in which all source tree datasets were obtained by sampling randomly within the full dataset. Here we describe the simulation methodology in brief, for details see [23].

#### Step 1: Generate model trees

We generated trees with 100, 500 and 1000 leaves (taxa) under a pure birth process, deviating these from ultrametricity (the molecular clock hypothesis). We generated 30 datasets for each 100- and 500-taxon model condition, and 10 datasets for each 1000-taxon model condition.

#### Step 2: Evolve gene sequences down the model tree

We first determined the subtree within the model tree for which each gene would be present, using a gene "birth-death" process (gene gain and loss); this produced missing data patterns that reflect biological processes. Each gene was then evolved down its subtree under a General Time Reversible process with rates for sites drawn from a Gamma plus Invariable distribution (GTR+Gamma+I) [24]), using a variety of GTR matrices estimated for different biological datasets (see Appendix [Additional file 1]).

#### Step 3: Dataset production

We selected (1) datasets of genes to estimate trees on specific clades (rooted subtrees) within the tree and (2) datasets of genes to form the scaffold tree. We selected three genes for each clade dataset, and four genes for each scaffold dataset. Each model condition is indicated by the number of taxa in the model tree and by the density of the scaffold dataset, which is the percentage of the entire taxon set in the scaffold dataset, with scaffold densities ranging from 20% to 100%. We generated two types of source tree dataset profiles: those containing only scaffolds, and those containing one scaffold and several clade-based datasets (as described earlier).

#### Step 4: Estimation of source trees

We used RAxML [25], one of the most accurate ML phylogeny estimation methods.

#### Step 5: Estimation of the supertrees

We used MRP, using a very effective heuristic search technique called *the Ratchet *[26] (see Appendix [Additional file 1] for commands used). This returns a set of trees, each of which has the best (found) score; we then compute the greedy consensus (gMRP) tree for this set. The greedy consensus is a refinement of the majority consensus, and thus contains all the bipartitions present in more than half the input trees; it is a common consensus method, and in our experiments produces the most accurate supertrees when applied to results produced by the Ratchet. We also computed supertrees based upon three ways of encoding the source trees as sets of quartet trees and then applying QMC, as described above. Finally, we computed supertrees using five other methods: Q-Imp, RFS, MinFlip, SFIT, and PhySIC (See Appendix [Additional file 1] for details on software and commands used).

Because MinFlip, RFS, and PhySIC require that the source trees be rooted, we rooted each source tree at the midpoint of the longest leaf-to-leaf path (a standard method for rooting trees when there is no outgroup available) before passing the source trees to these three methods.

#### Step 6: Performance evaluation

Topological error for each estimated supertree was measured as follows. We represented each tree *T *on leaf set *S *by the set ∑(*T*) of bipartitions induced on the leaf set, one bipartition for each internal edge in the tree. If *T *is an estimated supertree and *T*_0 _is the true (model) tree, then the *false positive rate *is , and the *false negative rate *is .

We also computed the total topological distance of each supertree to its source trees. To do this, we restricted the supertree to the subset of taxa for each source tree, and then computed the topological distances between the two trees. We computed the following three distance measures for each supertree *T *to its source tree profile .

**Sum-FN**, defined as follows: Sum-, where *FN*(*T*, *t*) is the number of edges in *t *that do not appear in *T*, and , where *m_t _*is the number of internal edges in *t*.

**Sum-FP **and **Sum-RF**, defined similarly, with *FP*(*T*, *t*) and *RF*(*T*, *t*) replacing *FN*(*T*. *t*), respectively. *FP *denotes the false positive distance and *RF *denotes the Robinson-Foulds ("bipartition") distance. The false positive distance between a supertree *T *and a source tree *t *in the profile  is the number of edges in *T *that do not appear in *t*. The Robinson-Foulds error rate is the average of the FP and FN error rates.

Each distance measure was normalized by the number of edges (bipartitions) in the relevant tree (the model tree for false negatives, and the estimated tree for false positives), to produce error rates between 0 and 1. Note that if the supertree and all source trees are binary, then *RF*(*T*, *t*) = 2*FN*(*T*, *t*) = 2*FP*(*T*, *t*), and after normalization all three distances are equal. When the estimated trees are not binary, the RF distance is biased in favor of unresolved trees [27]. Our source trees were generally fully binary or nearly fully binary. With the exception of PhySIC, the supertree methods we studied produced either fully resolved, or almost fully resolved supertrees. PhySIC is highly conservative and therefore tended to produce highly unresolved trees. Consequently, PhySIC tended to have very low false positive rates at the expense of having very high false negative rates. In our results, we, therefore, show false negative error rates, since except for PhySIC, the relative performance of the different supertree methods does not depend upon the error metric used. This allows us to provide a more nuanced evaluation than would be possible with RF. We calculated average error rates and standard error for each model condition. However, because QMC failed to return trees on some inputs, we restricted our results to datasets for which all the reported methods returned trees. This reduced the number of replicates for some model conditions. We also recorded the running time and space usage of each method on each dataset. Because the analyses were run under Condor (a distributed software environment [28]), running times are approximate (particularly for the larger datasets) and are larger than if they had been run on a dedicated processor.

## Results

### Exploring QMC under various quartet encodings

**Figure 1:**
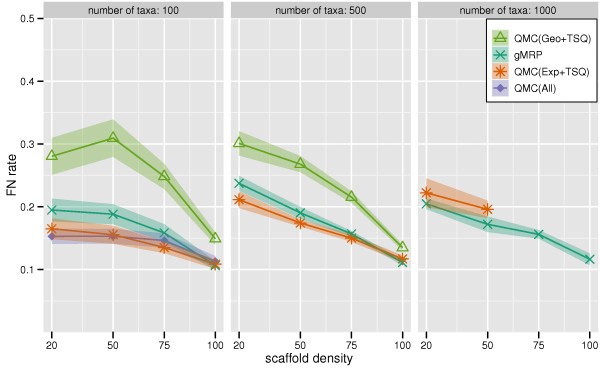
**Scaffold density vs. QMC-based and MRP FN rate**. False Negative (FN) error rates and error bars of QMC variants and gMRP on mixed source tree datasets with 100, 500, and 1000 taxa, as a function of the scaffold density. Points are graphed for a method if it had at least ten datasets (or four datasets, for the 1000-taxon model conditions) that completed in common with all other methods.

We show FN rates of QMC variants and gMRP on mixed datasets in Figure 1. On the mixed 100-taxon datasets, QMC(All) and QMC(Exp+TSQ) were essentially tied as the best methods, followed by gMRP. QMC(Geo+TSQ) had worse accuracy. Furthermore, QMC(All) and QMC(Exp+TSQ) had the greatest advantage over gMRP for the sparse scaffold cases. On a large number of the 500- and 1000-taxon datasets, many of the QMC variants failed to complete, indicating that computational issues can limit QMC's utility. On the 500-taxon datasets for which QMC(Exp+TSQ) could be run, it produced topologically more accurate trees than gMRP, providing the biggest advantage on the sparse scaffold datasets. For the 1000-taxon datasets, gMRP outperformed all the QMC variants that completed. However, most QMC variants failed to return trees on most inputs.

### Comparing QMC(Exp+TSQ) to other supertree methods

**Figure 2:**
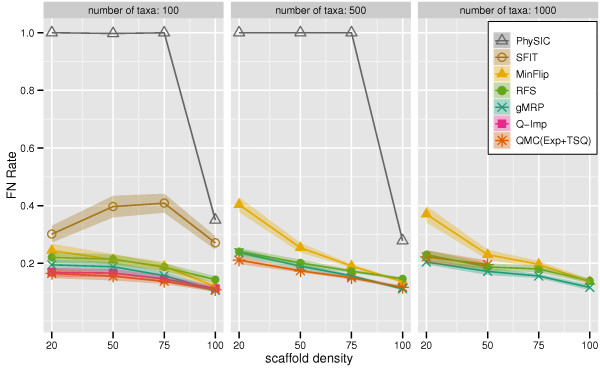
**Scaffold density vs. supertree method FN rate**. False Negative (FN) error rates and error bars of gMRP, SFIT, MinFlip, RFS, PhySIC, Q-Imp, and QMC(Exp+TSQ) on mixed source tree datasets with 100, 500, and 1000 taxa, as a function of the scaffold density. Points are graphed for a method if it had at least ten datasets (or four datasets, for the 1000-taxon model conditions) that completed in common with all other methods.

**Figure 3:**
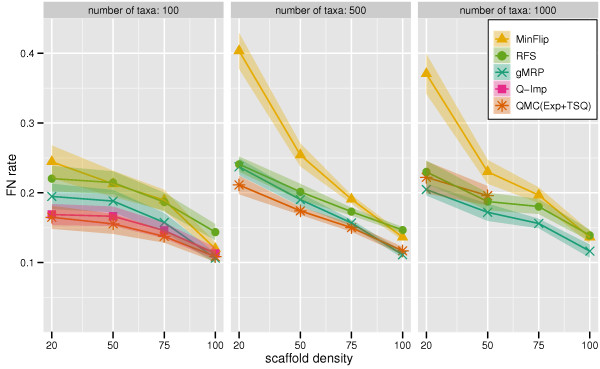
**Scaffold density vs. 4 best supertree methods' FN rate**. Topological error rates on mixed datasets, without PhySIC and SFIT (which had higher error rates). We report False Negative (FN) rates (means with standard error bars) for gMRP, MinFlip, RFS, Q-Imp, and QMC(Exp+TSQ), as a function of the scaffold density, for 100-, 500-, and 1000-taxon model conditions.

We report FN rates in Figure 2 (all methods) and Figure 3 (omitting PhySIC and SFIT). All six non-QMC-based supertree methods could be run on the 100-taxon datasets, but some failed to run on the larger datasets. We, therefore, show results for all seven methods on the 100-taxon datasets, but only five methods on the 500-taxon datasets (where SFIT and Q-Imp failed to run, due to computational limitations), and only four methods on the 1000-taxon datasets (where we did not try to run PhySIC, since it had poor topological accuracy and was computationally intensive for the 500-taxon datasets). As noted above, QMC(Exp+TSQ) failed to run on some datasets, so we again only report results for those datasets on which all reported methods were able to run.

On the 100-taxon datasets, QMC(Exp+TSQ) and Q-Imp both had higher accuracy than gMRP, except on the 100% scaffold datasets, where they were equal. On the 500-taxon datasets, QMC(Exp+TSQ) had a slight advantage over gMRP on the sparse scaffold datasets, but essentially the same accuracy on datasets with the two densest scaffolds. On the 1000-taxon datasets, gMRP had an advantage over QMC(Exp+TSQ), and QMC(Exp+TSQ) failed to run on the denser scaffold datasets (large source trees caused QMC to fail due to computational reasons). On all these model conditions, gMRP had higher accuracy than the remaining methods. PhySIC gave by far the worst results, producing completely unresolved trees except when the scaffold density was 100%, at which point it produced results that were still worse than the other methods.

### Evaluating the impact of taxon sampling strategies

**Figure 4:**
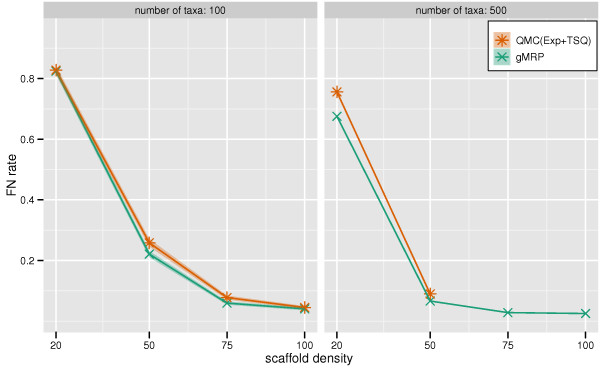
**Scaffold density vs. supertree method FN rate on all-scaffold data**. Topological error rates on 100- and 500-taxon all-scaffold datasets. We report False Negative (FN) rates (means with standard error bars) for QMC(Exp+TSQ) and gMRP as a function of the scaffold density.

Supertree studies differ not only in the methods used to combine source trees into a tree on the full set of taxa, but also in how the source tree datasets are produced, and in particular how densely sampled these source trees are. On datasets that have only one scaffold, the accuracy of all supertree methods suffer as the density of the scaffold decreases, a trend that was also observed by Swenson et al. [23] (see Figures 1, 2, 3). Figure 4 shows the results of an experiment in which we sought to evaluate the impact of the density of taxon sampling within source trees on the accuracy of the produced supertree for 100- and 500-taxon all-scaffold datasets; we did not generate 1000-taxon all-scaffold datasets, and therefore did not analyze such datasets using any supertree methods, due to the running time required to estimate dense scaffolds for such datasets. We compared the topological accuracy of supertrees estimated on all-scaffold datasets with those from mixed-datasets (datasets having one scaffold source tree with the remaining source trees being clade-based).

We found that the density of taxon sampling in the source trees in all-scaffold datasets has a strong effect on supertree accuracy, particularly at low scaffold densities. When the source trees were all based upon sparsely sampled scaffold datasets, the FN error rates were high for both gMRP and QMC(Exp+TSQ), and much higher than when most of the source trees were clade-based. In addition, there was only a slight advantage obtained by using gMRP over QMC(Exp+TSQ). We also examined the performance of QMC(All) on these all-scaffold datasets (data not shown), and saw that it performed poorly, failing to return trees on most of the datasets. For example, on the 100-taxon all-scaffold datasets, QMC(All) returned a tree on none of the 20% scaffold datasets, two of the 50% scaffold datasets, on eleven of the 75% scaffold datasets and on four of the 100% scaffold datasets. However for those datasets for which it did return trees, they were less accurate than QMC(Exp+TSQ). Because QMC(All) returned trees for very few datasets, we did not include data for it in Figure 4.

We also analyzed all-scaffold datasets with 500 taxa and observed the same trends: gMRP and QMC(Exp+TSQ) both had poor accuracy on the sparse scaffold model conditions, and-when both could be run-had comparable accuracy. In addition, we note that QMC(Exp+TSQ) could not be run on the dense 500-taxon scaffold conditions, and QMC(All) successfully completed on only two of the 20% scaffold datasets and none for denser scaffolds.

In summary, the general performance on the all-scaffold datasets showed that whenever the scaffold density was low, the absolute topological error rates were very high. Furthermore, on these all-scaffold datasets, QMC variants rarely returned trees. On datasets for which they did return trees, the best QMC analyses were quite close to those of MRP.

### Using topological distances to source trees as a proxy for topological accuracy

For biological datasets, the true tree is not available, so evaluations of supertree accuracy have tended to use average or total topological distance to the source trees (see, for example, [14,15]). Is this a good proxy for the quality of the supertree?

To address this question, we examined how closely Sum-FN, Sum-FP, and Sum-RF were correlated with the FN, FP and RF rates, respectively. We calculated Spearman rank-correlations for each of the 100-taxon simulated datasets for the six supertree methods that consistently performed reasonably well (MinFlip, MRP, Q-Imp, QMC(All), QMC(Exp+TSQ), and RFS). Table 1 gives the correlations for the 100-taxon model conditions. The statistics were calculated this way to test whether the rank-order of the topological distances to source trees correlated strongly with the true rank-order of the supertrees, in terms of topological accuracy with respect to the true tree. We found the degree of correlation was largely independent of the choice of topological distance to the source trees and absolute supertree error because the true supertrees were fully resolved and all the estimated supertrees were either fully resolved or nearly fully resolved. We, therefore, focus on the correlation between SumFN (topological distance to the source trees) and FN (topological distance to the true tree).

**Table 1:** Correlation between topological distance to source trees and topological error rates

Scaffold Density	Optimality Criterion	FN		FP		RF	
				
		Mean	Range	Mean	Range	Mean	Range
	SumFN	0.401	-0.890, 0.939	0.376	-0.890, 0.926	0.391	-0.890, 0.926
25%	SumFP	0.421	-0.890, 0.939	0.421	-0.890, 0.926	0.426	-0.890, 0.926
	SumRF	0.406	-0.890, 0.939	0.395	-0.890, 0.926	0.406	-0.890, 0.926

	SumFN	0.544	-0.203, 1.000	0.536	-0.348, 0.971	0.541	-0.203, 0.971
50%	SumFP	0.546	-0.143, 1.000	0.539	-0.257, 0.971	0.543	-0.143, 0.971
	SumRF	0.546	-0.143, 1.000	0.539	-0.257, 0.971	0.543	-0.143, 0.971

	SumFN	0.593	-1.000, 0.986	0.589	-1.000, 0.986	0.591	-1.000, 0.986
75%	SumFP	0.593	-1.000, 0.986	0.589	-1.000, 0.986	0.591	-1.000, 0.986
	SumRF	0.593	-1.000, 0.986	0.589	-1.000, 0.986	0.591	-1.000, 0.986

	SumFN	0.447	-0.789, 1.000	0.447	-0.789, 1.000	0.447	-0.789, 1.000
100%	SumFP	0.447	-0.789, 1.000	0.447	-0.789, 1.000	0.447	-0.789, 1.000
	SumRF	0.447	-0.789, 1.000	0.447	-0.789, 1.000	0.447	-0.789, 1.000

Results of Spearman rank-order correlations of SumFN, SumFP, and SumRF with the true FN, FP, and RF measures of supertrees estimated using six supertree methods.

The results show that using the distance of a supertree from its source trees is not a reliable optimality criterion for assessing the topological accuracy of the supertree. In no case was the correlation with true accuracy for a given scaffold density greater than 60%. Furthermore, some datasets had a strong negative correlation between SumFN and the true quality of the supertrees, making the optimality criterion positively misleading in those cases.

### Scalability

**Figure 5:**
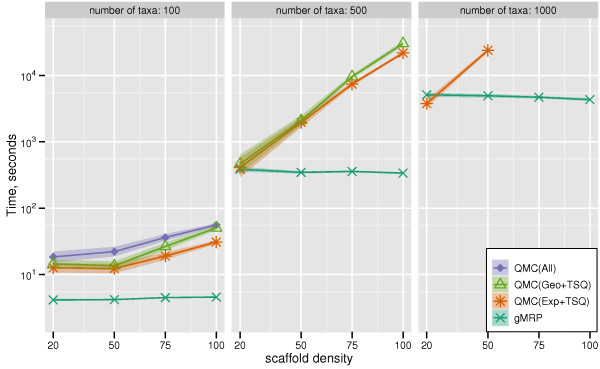
**Scaffold density vs. QMC-based and MRP running times**. Running times (in seconds) of QMC supertree methods and gMRP on mixed datasets; the y-axis is given with a logarithmic scale.

**Figure 6:**
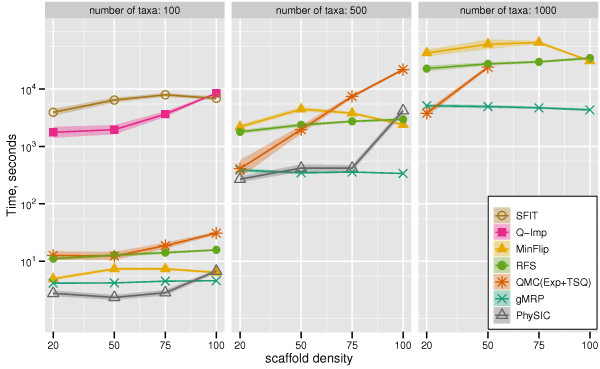
**Scaffold density vs. supertree method running times**. Running times (in seconds) of supertree methods on mixed datasets; the y-axis is given with a logarithmic scale.

We compared the running time of all supertree methods on simulated data. Figure 5 gives the results for the QMC variants and gMRP, and Figure 6 gives results for gMRP, QMC(Exp+TSQ), and the other (non-QMC-based) supertree methods.

Supertree methods on the simulated datasets showed some differences in running times. First, gMRP was faster than the accurate QMC variants for most of the model conditions, and the degree of improvement ranged from very small (a few seconds) to several hours. In general, we saw that profiles with large source trees were particularly computationally intensive for QMC(Exp+TSQ) and QMC(All), and that for such datasets, gMRP had a running time advantage.

We note that the running times of QMC(All), QMC(Geo+TSQ), and QMC(Exp+TSQ), were strongly impacted by the size of the source trees, since each four-tuple of taxa must be examined to produce the quartet trees. Thus, for large source trees, we expect these three QMC methods to suffer computationally, just because of the number of quartets that are examined. In addition, needing to store a large set of quartets also impacts the memory requirements of the method. Note that the number of quartets produced by each encoding varied dramatically, with QMC(Geo+TSQ) by far producing the fewest, followed by, QMC(Exp+TSQ), then with many more, and finally by QMC(All) (Table 2). On the other hand, we also observed that QMC(All) will not run on some datasets even though QMC(Exp+TSQ) may run, and vice-versa. Thus, it is possible that improved QMC software could increase the scope of problems on which the method can be used and increase the reliability of the method.

**Table 2:** Number of quartets

	**100 taxa**				**500 taxa**			
Methods	(20%)	(50%)	(75%)	(100%)	(20%)	(50%)	(75%)	(100%)
QMC(Geo+TSQ)	2,033	2,102	2,759	4,046	65,363	102,255	223,174	487,242
QMC(Exp+TSQ)	18,799	19,704	25,388	34,432	268,335	433,546	694,134	1,088,577
QMC(All)	2,738,798	2,652,543	3,712,832	6,362,857				

Average number of quartets input to QMC on *mixed *datasets; scaffold densities are shown in parentheses.

## Conclusions

This study makes several important contributions. First, and most importantly, we show that MRP is no longer the sole "method to beat," since both QMC(Exp+TSQ) and Q-Imp produce more accurate supertrees than MRP under many realistic conditions. On the other hand, MRP does outperform all the other supertree methods we tested and remains the most accurate method that can be consistently run on profiles that contain large source trees. Overall, we have shown that improved supertree methods are possible and that an effort should be made to produce scalable and robust implementations of the most accurate supertree methods. The computational limitations of QMC(Exp+TSQ) and Q-Imp result from the fact that each of these methods produces a quartet encoding of the source trees. Scalable implementations of these methods will require *not *using all the quartets in these encodings, as such approaches simply will fail on large datasets.

The second important contribution of the study is the finding that the total topological distance of a supertree to its source trees can be a very poor optimality criterion, and that these distance measures can only provide reliable comparisons between supertrees that have very different total topological distances. This observation has several consequences for supertree analyses. First, directly trying to optimize the total topological distance of supertrees to their source trees is not likely to produce the most accurate trees, since better trees are being produced through other means. Secondly, because the true tree is not known for biological supertree datasets, it is difficult to evaluate supertree methods using biological datasets. Finally, previous studies that have explored performance of supertree methods using total topological distance to the source trees need to be revisited.

Our study also shows that supertree analyses are very much impacted by the strategies used to define the source tree datasets, with sparse "all-scaffold" datasets resulting in generally much lower accuracy supertrees than when the source trees are primarily based upon dense sampling within clades. This final observation has significant consequences for systematic studies, and for attempts to assemble the Tree of Life.

Finally, our conclusions are clearly based upon the conditions of this experiment, in which the source trees were reasonably, but not extremely, accurate. (If all the source trees had been accurate, then most supertree methods would have performed well, provided that the source trees had good overlap. In that case, supertrees based upon either MRP or minimizing the topological distance to the source trees would be guaranteed to return the true tree as one of the solutions.) Most source trees are likely to have some error when using real biological datasets for at least two reasons. First, alignments must be estimated, and these can be difficult for some datasets with many insertions and deletions. (By contrast, in our simulation study, sequence evolution occurred without indels, and so the true alignment was known). Second, while maximum likelihood can be a very accurate phylogeny estimator when the sequences evolve under the model assumed in the ML software, true biological datasets do not evolve under the idealized conditions reflected in even the most complex DNA sequence evolution models used in this experiment. Therefore, phylogenies estimated under ML for real datasets are likely to have more error than we observed in these simulations. How supertree methods will respond to increased error in source trees is a subject for further study.

## Declaration of competing interests

The authors declare that they have no competing interests.

## Authors' contributions

MSS designed and performed the simulation study, and drafted the manuscript. RS assisted in simulation study and data analyses and created the figures. TW conceived the study, assisted in the design and analysis of the simulation study, and helped draft the manuscript. CRL assisted in the design and analysis of the simulation study, performed the statistical study comparing topological distances to source trees to topological error, and revised the manuscript. All authors read and approved the final manuscript.

## Supplementary Material

Additional file 1 **Appendix**. The appendix includes the commands used to perform the simulation study. Click here for file
