# Supplementary material for: An experimental study of Quartets MaxCut and other supertree methods
Source: Algorithms Mol Biol. 2011 Apr 19;6:7. doi: 10.1186/1748-7188-6-7 (PMC3101644; doi:10.1186/1748-7188-6-7)
Supplement: Additional file 1 — Appendix. The appendix includes the commands used to perform the simulation study. [file 1748-7188-6-7-S1.PDF]

Appendix: Details of commands used in data generation

Commands for model tree generation

The commands given to r8s, to construct initial model trees are as follows:
simulate diversemodel=yule_c T=1
ntaxa= *desired_number_of_taxa* nreps=1 seed= *random_seed*
speciation=1 charevol=yes ratemodel=normal startrate=1 changerate=0.05
infinite=yes minrate=0.05 maxrate=8;

Commands for gene sequence generation

The commands given to Seq-Gen for each of the three model conditions are as follows:

Angiosperm data set: -on -z 500 -mGTR -a 0.5 -i 0.2
-r 1.54755 3.67531 1.86115 0.93047 4.53303 1.0
-f 0.223269 0.206748 0.256568 0.313414 -s *mean*

Nematode data set: -on -z 500 -mGTR -a 0.362026 -i 0.273196
-r 1.24284 3.47484 0.48667 1.07118 4.38510 1.0
-f 0.300414 0.191363 0.196748 0.311475 -s *mean*

rbcL data set: -on -z 500 -mGTR -a 0.397524 -i 0.101878
-r 1.09397 3.12811 0.35141 1.55972 3.64704 1.0
-f 0.320128 0.176726 0.167462 0.335683 -s *mean*

Command for source tree inference

RAxML commands used in ML analyses are as follows:
raxmlHPC -s <phylip_alignment_file> -n <output_suffix> -m GTRMIX

Commands for supertree estimation

MinFlip (version 2.2): To run MinFlip, one must first create a Nexus format file of the matrix representation of the set of source trees. This Nexus file is the input to the MinFlip heuristic.
HeuristicMFT2 --off <matrix_rep_of_source_trees_file> <output_file>

MRP (Paup 4.0b10): As with MinFlip, MRP requires the matrix representation of the set of source trees.

Parsimony ratchet comands. The PAUP* block used to perform the parsimony ratchet search is as follows:
<matrix_rep_of_source_trees>
begin paup;
set autoclose=yes warntree=no warnreset=no
notifybeep=no monitor=yes taxlabels=full;
log file=<log_file> replace;

```
set criterion=parsimony;
pset collapse=no;
                    [!][!*** Replicate 0 (initial tree) ***]
hsearch addseq=random nreps=1 rseed=<random_seed>
swap=TBR multrees=no dstatus=60;
savetrees file=<tree_file> format=altnex replace;
savetrees file=<nexus_tree_file> format=nexus replace;
    Then, for each integer i from 1 to n, [!][!*** Replicate i ***]
weights  list of character weights (chosen as described in Methods section);
hsearch start=current swap=TBR multrees=no dstatus=60;
weights 1:all;
hsearch start=current swap=TBR multrees=no dstatus=60;
savetrees file=<tree_file> format=altnex append;
savetrees file=<nexus_tree_file> format=nexus append;
                    [!][!*** Determining consensus trees ***]
set MaxTrees=201;
gettrees file=<tree_file> allblocks=yes warntree=no;
set criterion=parsimony;
condense collapse=no deldupes=yes;
filter best=yes;
contree all / majrule=yes strict=no le50=yes treefile=<mMRP_file> replace;
savetrees file=<tree_file> replace=yes format=altnex;
log stop; end; quit warntsave=no;
```

Q-imputation (version 1.0):

```
quartet log=q_imp.log algorithm=simple output=qimp_output
tree=<source_trees_file>
```

QMC (version 1.0) The code available for running QMC requires that the taxa names be non-negative integers (i.e. involve only digits not letters or other characters). Once this translation is made QMC takes as input a file containing the set of selected quartet trees. We used the following command:

```
find-cut qrtt=<quartet_file>  > <output_file>
```

PhySIC (version 1.2):

```
physic -s <source_trees_file> -t 0 > <output_file>
```

RF-Supertree (version 1.0):

```
RF-SPR.linux -i <source_trees_file> -o <output_file>
```

SFIT (Clann 3.1.3):

```
clann <source_trees_file>
set criterion sfit
hs savetrees <output_file>
```
